# SWEET DREAMS INTERRUPTED: HOW COVID-19 CHANGED THE LANDSCAPE OF SLEEP SERVICES UTILIZATION ON AGING POPULATION

**DOI:** 10.1093/geroni/igad104.2314

**Published:** 2023-12-21

**Authors:** Javad Razjouyan, Samuel T Kuna, Amir Sharafkhaneh

**Affiliations:** Baylor College of Medicine, Houston, Texas, United States; Corporal Michael J. Crescenz VA Medical Center, Philadelphia, Pennsylvania, United States; Baylor College of Medicine, Houston, Texas, United States

## Abstract

We evaluated the impact of the pandemic on sleep test utilization, i.e., home sleep apnea testing (HSAT) and in-laboratory polysomnography (PSG), among aging veterans. We conducted an observational before and during the pandemic study using the national VHA. Electronic medical records from 01/2019 to 09/2022 were gathered for all veterans born with age 50+ by 10/2022. HSAT and PSG utilization were defined based on Current Procedural Terminology codes. Following intervals defined as pre-pandemic (PRE-P, 01/2019-02/2021, 14months), Pandemic-moratorium (PMor, 4/2020-6/2020, 3months), pandemic-pre-vaccination opening (PnoVax, 7/2020-12/2020, 6months), pandemic vaccination (P-Vax, 1/2021-5/2021, 5months), and pandemic post vaccination (P-PVax, 06/2021-09/2022, 15months). We compared the means using univariant analysis. Over the 45 months, 247,817 HSAT and 123,456 PSG were performed. In HSAT, compared to PRE-P (5,669visit/month) as a reference, we observed a significant drop in P-Mor (-70%,P< 0.001), P-noVax (-7%,P=0.166), P-Vax (+9%,P=0.067). However, P-PVax did not differ significantly in average monthly HSAT compared to PRE-P (+6%,P=0.103). In PSG, compared to PRE-P (3,994visit/month) as a reference, we observed a significant drop in P-Mor (-92%,P< 0.001), P-noVax (-61%,P< 0.001), P-Vax (-44%,P< 0.001), and P-PVax (-32%,P< 0.001). PSG per month did not recover to pre-pandemic levels, while the HSAT per month increased, but not to a level that compensated for the reduction in PSG. The pandemic markedly impacted the sleep testing service usage in aging veterans. While HSAT was resilient and increased during the pandemic, the increase did not offset the shift away from PSG. Future research is needed to better understand the reasons for this change in clinical practice among aging populations.

